# The human-initiated model of wolf domestication – An expansion based on human-dingo relations in Aboriginal Australia

**DOI:** 10.3389/fpsyg.2023.1082338

**Published:** 2023-05-02

**Authors:** Adam Brumm, Mietje Germonpré, Loukas Koungoulos

**Affiliations:** ^1^Australian Research Centre for Human Evolution, Griffith University, Brisbane, QLD, Australia; ^2^Royal Belgian Institute of Natural Sciences, Brussels, Belgium; ^3^School of Humanities, The University of Sydney, Sydney, NSW, Australia; ^4^College of Asia and the Pacific, The Australian National University, Canberra, NSW, Australia

**Keywords:** Late Pleistocene, wolf domestication, pet-keeping, dingoes, Aboriginal Australians

## Abstract

The historically known relationship of interspecies companionship between Aboriginal foraging communities in Australia and free-ranging dingoes provides a model for understanding the human-canid relations that gave rise to the first domesticated dogs. Here, we propose that a broadly similar relationship might have developed early in time between wild-living wolves and mobile groups of foragers in Late Pleistocene Eurasia, with hunter-gatherers routinely raiding wild wolf dens for pre-weaned pups, which were socialized to humans and kept in camp as tamed companions (“pets”). We outline a model in which captive wolf pups that reverted to the wild to breed when they were sexually mature established their territories in the vicinity of foraging communities — in a “liminal” ecological zone between humans and truly wild-living wolves. Many (or most) of the wolf pups humans took from the wilderness to rear in camp may have derived from these liminal dens where the breeding pairs had been under indirect human selection for tameness over many generations. This highlights the importance of the large seasonal hunting/aggregation camps associated with mammoth kill-sites in Gravettian/Epigravettian central Europe. Large numbers of foragers gathered regularly at these locations during the wild wolf birthing season. We infer that if a pattern of this kind occurred over long periods of time then there might have been a pronounced effect on genetic variation in free-ranging wolves that denned and whelped in the liminal zones in the vicinity of these human seasonal aggregation sites. The argument is not that wolves were domesticated in central Europe. Rather, it is this pattern of hunter-gatherers who caught and reared wild wolf pups gathering seasonally in large numbers that might have been the catalyst for the early changes leading to the first domesticated dogs — whether in western Eurasia or further afield.

## Introduction

The dog (*Canis familiaris*) is widely regarded by scientists as our oldest domesticated animal (Germonpré et al., 2009; Larson and Fuller, 2014; Perri et al., 2021; Shipman, 2021; Bergström et al., 2022). Genetic evidence suggests that dogs probably descend from one or possibly a few now-extinct Eurasian wolf populations (Thalmann et al., 2013; Freedman et al., 2014; Frantz et al., 2016; Bergström et al., 2020; Gojobori et al., 2021; Bergström et al., 2022), although with behavior comparable to that of extant gray wolves (*C. lupus*) (Mech and Janssens, 2022). The first widely accepted skeletal remains of *C. familiaris* are from Magdalenian sites in Spain, Germany and Switzerland, and date to around 17,000–14,000 years ago (ka) (Nobis, 1986; Napierala and Uerpmann, 2012; Street et al., 2015; Hervella et al., 2022). On the basis of this evidence it is broadly agreed that, prior to this time, a non-sedentary population of hunter-gatherers had entered into a domestic relationship with wolves in some part of the Eurasian landmass (including eastern Asia) (Perri, 2016; Perri et al., 2021; Bergström et al., 2022). Age estimates of up to 40 ka for the beginnings of wolf domestication have been proposed on the basis of genetic (Botigué et al., 2017), morphological (Germonpré et al., 2009, 2012, 2015a), and isotopic data (Germonpré et al., 2009; Bocherens et al., 2015).

There are two competing hypotheses of wolf domestication: (1) wolves self-domesticated by adapting genetically to anthropogenic environments as commensal scavengers (Coppinger and Coppinger, 2001; Zeder, 2012; Larson and Fuller, 2014; Morey and Jeger, 2015; Hare, 2017; Range and Marshall-Pescini, 2022a); and (2) wolf domestication emerged from the process of hunter-gatherers taking wolf pups from wild dens and hand-raising them as companions (“pets”) (Serpell, 1989, 2021; Müller, 2005; Germonpré et al., 2015b, 2018, 2021a; Mech and Janssens, 2022). It is our contention that the latter, human-initiated, cross-species adoption hypothesis provides the most plausible account of how dogs were domesticated from wolves. The main strength of the hypothesis is that it generally fits with what is known about the behavior of modern free-roaming wolves (Serpell, 2021; Mech and Janssens, 2022). It is also consistent with the well-documented propensity of our species to rear and keep nonhuman animals as pets (Galton, 1865; Batchelor, 1901; Shirokogoroff, 1935; Serpell, 1989, 1996; Cormier, 2003; Serpell, 2021). In contrast, opponents of the self-domestication hypothesis have identified a number of flaws in its logic that are difficult to reconcile with the current knowledge of wolf biology and behavioral ecology (Serpell, 2021). In particular, critics argue that the mobile foraging groups of Late Pleistocene Eurasia are unlikely to have produced enough edible waste at their habitation sites to attract and sustain a large population of scavenging wolves (Lupo, 2019; Serpell, 2021). And even if they did, foragers are unlikely to have tolerated the presence of commensal wild wolves that had become habituated to humans and so may have engaged in predatory attacks against them (Koler-Matznick, 2002; Germonpré et al., 2018; Lupo, 2019; Serpell, 2021).

However, although in our view cross-species adoption provides a more plausible account for the origins of domestic dogs, there are some challenges with this “origin story” that require resolution before pup-raising can be accepted as the most credible general explanation for the beginnings of wolf domestication. We reach this conclusion based on available insights into the complex relationship between Aboriginal people and the wild-living canid of Australia, the dingo (*Canis dingo*). Australia is host to the only mobile foraging communities whose ubiquitous practice of taking the pups of free-ranging canids from their dens and raising them as companion animals is relatively well attested in the historical record (Meggitt, 1965; Smith and Litchfield, 2009; Balme and O’Connor, 2016; Brumm, 2021; Koungoulos, 2021). In this paper we critically investigate the human-initiated hypothesis of wolf domestication, and expand on this theory based on a detailed consideration of human-dingo relations. We specifically aim to address two key points: (1) the conceptualization of how early hunter-gatherers in Eurasia might have interacted with socialized wolves that had reached breeding age; and (2) how wild-caught, human-raised, socialized wolves could have become reproductively isolated from wild populations — giving rise to early dogs.

## The human-initiated model of wolf domestication

The basic premise of this hypothesis is that hunter-gatherers in Late Pleistocene Eurasia adopted very young wolf pups and hand-reared them for several reasons or motivations (Galton, 1865; Serpell, 1989, 2021; Sauer, 1952:31; Zeuner, 1954:331; Müller, 2005; Germonpré et al., 2018; Mech and Janssens, 2022). Scholars propose that the earliest modern humans to spread into Eurasia (the so-called Aurignacian “culture”; ~42–34 ka) routinely took pre-weaned wolf pups from wild dens and kept some to hand-rear in their home communities (Germonpré et al., 2018, 2021a). The underlying motivations may have been the animistic cosmology of Late Pleistocene foraging societies, cultural traditions (i.e., ritual practices), the need to obtain bodily products of these animals, and a desire for inter-species companionship (Serpell, 1989; Germonpré and Hämäläinen, 2007; Germonpré et al., 2018; Kotrschal, 2018). Upper Paleolithic people may have also occasionally adopted and raised the orphaned young of other non-human animal species (Serpell, 1989), including other large carnivores (e.g., bears), as well as smaller canids like foxes (Germonpré et al., 2018; Baumann et al., 2021). As is suggested by Upper Paleolithic rock art and portable artworks, it seems that people during this time period placed particular value on the personal interaction with individual animals (Porr and de Maria, 2015; Lequellec, 2022). The natural sociality of Pleistocene wolves and their standing variation in behavior, along with other characteristics such as adaptable lifestyle and tolerance for inbreeding, the behavioral and social parallels between wild wolves and humans — both living in families, and ecological overlap — may have predisposed this wild canid taxon, in particular, to enter into an early domestic relationship with our species following the human-initiated, pup-raising pathway (Germonpré et al., 2018, 2021a; Kotrschal, 2018; Mech and Janssens, 2022; Range and Marshall-Pescini, 2022a,b). On the other hand, we have limited knowledge of the nature (and closeness) of early human interactions with other potential companion species (e.g., wild pigs; see Brumm, 2023).

It is reasoned that Late Pleistocene women and children are likely to have played a central role in hand-raising young adopted wolves and other juvenile animals taken from the wild (Koungoulos, 2021; Germonpré et al., 2021a). In addition, wolf pup culling or capturing at wild dens could have been practiced to reduce interspecific competition for prey (Farnell et al., 2005), comparable to a custom Central Asian herders traditionally undertake to protect herds of domestic ungulates (Heptner et al., 1998; Lescureux, 2007), or to avoid predators raiding occupied camps for food. The assumption is that Late Pleistocene humans removed wild-born wolf pups from their dens when the animals were less than about 2–3 weeks old. In modern wolf-raising and socialization programs, pups are usually taken from natal dens when they are 8–10 days old (Klinghammer and Goodmann, 1987; Range and Virányi, 2014). At this point the pups are still blind, the mother leaves the den more often, and she is more easily distracted (Packard, 2003). These individuals, from a developmental perspective, would have still been within the critical period of socialization during which wild wolf pups can potentially be socialized to humans (Klinghammer and Goodmann, 1987; Lord, 2013; Hall et al., 2015).

The implicit assumption here is that in Eurasia the captive wolf pups were selected by Late Pleistocene hunter-gatherers from a very young age for their human-friendly behavior, and potentially also for favored morphological features, such as particular coat colors that humans found aesthetically appealing (Germonpré et al., 2018, 2021a). Probably, humans choose for a specific segment of the wild wolf population (*cf.* Spengler, 2022). Indeed, recent studies have shown that human-directed attachment behaviors likely existed as standing variation within Pleistocene wolf populations (Hansen Wheat et al., 2022). Pups without the desired characters are likely to have been culled, either at the time of the den raid or at a later stage when individual pups’ temperament and demeanor could be more reliably assessed (Germonpré et al., 2018, 2021a). Concerning the latter, it is argued that the more sociable and playful pups would be favored over the warier, more reactive and less tractable ones. It is further assumed that captive pups that were not culled were subjected to an intensive process of socialization, in which cross-species wet nursing was key (Simoons and Baldwin, 1982). By feeding and nurturing the pups, humans took advantage of the natural sociality of wolves and intervened in the critical imprinting phase in their behavioral development (Germonpré et al., 2018, 2021a). This enabled adoptive human parents to form a close bond with their pups (Mech and Janssens, 2022), which were kept and cared for until they reached reproductive age (Germonpré et al., 2018, 2021a).

A commonly voiced objection to the human-initiated model for wolf domestication is that any pups born in camp to wild-caught, human-socialized wolves would still be genetically wild animals, and hence, the taming and socialization process must be repeated each new generation (Coppinger and Coppinger, 2001). Socialized wolves that have reached sexual maturity are strong and intimidating animals (Klinghammer and Goodmann, 1987). While the individual temperament of wolves varies widely (Packard, 2003), and it is not necessarily the case that hand-reared and human-socialized wolves are inherently dangerous as adults, some clearly are (e.g., Kolmården wildlife park incident in 2012); in modern settings they are typically housed in secure enclosures and handled with care for good reason (Klinghammer and Goodmann, 1987; Kubinyi et al., 2007; Ujfalussy et al., 2017). Individualized attachment bonds can still be maintained with wolves that have been hand-reared by people (Ujfalussy et al., 2017; Lenkei et al., 2020; Hansen Wheat et al., 2022). However, some socialized adult wolves have a predilection to subject even familiar humans to agonistic dominance tests (Klinghammer and Goodmann, 1987). They also tend to have a strong prey drive and some individuals may attack people displaying a debility (Klinghammer and Goodmann, 1987:53).

Germonpré et al. (2021a) contend that Upper Paleolithic people would have dealt with the threat to human safety by keeping mature she-wolves tethered or in some way immobile or incapacitated, such as muzzling the wolves, or tying them up in such a manner that the animals were unable to chew through the binding [as documented in Australia with dingoes (Nind, 1831)]; they then would have disposed of these animals once they had delivered their first litter, in order that they could adopt their pups. Mech and Janssens (2022), on the other hand, argue that early hunter-gatherers would have only remained in close association with socialized adult wolves that were able to co-exist peacefully with humans. This implies that intensive human selection against aggressive-predatory behavior in captive pups weeded out the more dangerous individuals. Under both scenarios, which could be end points of a continuum, new litters of captive wolf pups were subjected to the same process of human selection on behavior and/or morphology. The assumption is that a long-term pattern of artificial selection on subsequent generations progressively modified human-socialized wolves into divergent lineages of genetically domestic canids in different regions of Eurasia. Some of these lineages could be the earliest direct antecedents of modern-day dogs, while others became extinct (Serpell, 1989, 2021; Germonpré et al., 2015b, 2018; Mech and Janssens, 2022).

A key problem for the pet-keeping hypothesis is explaining *how* socialized wolves became reproductively isolated from surrounding populations of wild wolves (Serpell, 2021). Recently, Mech and Janssens (2022) proposed a novel approach to this problem. Drawing on their extensive knowledge of modern wild wolf behavior and biology, these researchers infer that when human-socialized wolves reached sexual maturity they would have commonly formed mated pairs (the basic social unit of the wolf world) with socialized individuals of the opposite sex (Mech and Janssens, 2022). They suggest that humans would have been motivated to regularly hand-feed these socialized breeding wolves in order to keep them dependent on humans. The socialized adult wolves, Mech and Janssens (2022) argue, are likely to have scent-marked the area around their human community’s campsite to establish it as their territory, and howled frequently to keep wild wolves away. They also contend that ‘wolves raised by humans and totally dependent on them for food would den closer to them’ (Mech and Janssens, 2022:7). These authors surmise that pregnant females gave birth to their pups within the bounds of human settlements, where food was available and they were protected by their human “pack.” Another possibility they raise is that socialized wolves frequently denned near human encampments (Mech and Janssens, 2022).

Hence, Mech and Janssens (2022) propose that by routinely provisioning hand-reared and human-dependent adult wolves, Late Pleistocene foragers were able to acquire any pups breeding pairs in this category may have sired, which they then hand-raised in camp. They stress that: ‘The key to this process is humans regularly feeding the wolves and continually keeping only those living peacefully and with the least trouble’ (Mech and Janssens, 2022:7). Notably, Mech and Janssens (2022) argue that their scenario differs markedly from the commensal scavenger theory, which posits that wild wolves only indirectly fed from humans (i.e., by scavenging from their occupation areas), and so would not have interacted closely with them (see also Germonpré et al., 2018, 2021a for similar characterizations).

Importantly, Mech and Janssens (2022) contend that in order for the socialized wolves to become reproductively isolated from their wild relatives (unsocialized wolves that lived independently of humans) it would be necessary (1) for mated pairs of human-dependent wolves to reuse the same dens each breeding season; and (2) for mobile groups of hunter-gatherers to return regularly to the same location where the socialized wolves were denning and whelping. The implication is that if both criteria were met then foragers would be more likely to procure the progeny of human-selected, hand-reared wolves. This is a critical factor, as it explains how human-raised wolves became isolated from the wild genetic pool.

These various extrapolations of the human-initiated model of wolf domestication are plausible from the perspective of modern wolf biology and behavior, at least according to Mech and Janssens (2022). A shortcoming of the model, however, is the lack of comparative ethnographic examples of relationships of this nature involving mobile groups of foragers and wild-living canids (Koungoulos, 2021). Here, therefore, we examine the plausibility of the pup adoption hypothesis for the origin of domestic dogs in the light of what it is known about human-dingo relations in Aboriginal Australia.

## The Australian dingo

**Figure 1:**
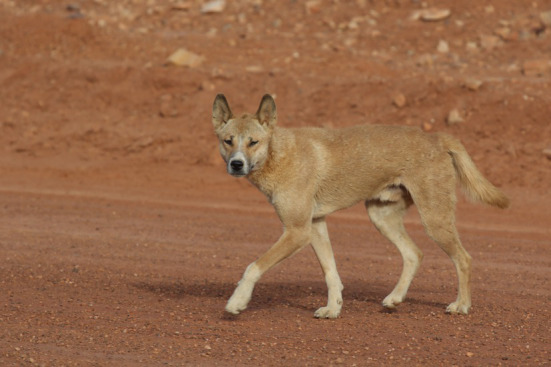
The Australian dingo (*Canis dingo*). Source: B. Smith. Reproduced with permission.

The wild or native “dog” of Australia is colloquially known as the dingo (Figure 1) (Crowther et al., 2014), although there is an ongoing disagreement over the taxonomic identity of these canids, especially the binomial nomenclature (Jackson et al., 2017). Modern humans have been in Australia for at least 50,000 years (Allen and O’Connell, 2020) and possibly much longer (Clarkson et al., 2017). However, archeological evidence suggests that ancestral dingoes were brought to northern Australia at some stage in late prehistory by an as yet unidentified group of seafaring hunter-gatherers (Balme et al., 2018). The earliest securely dated skeletal remains of the dingo in Australia date to 3,348–3,081 calibrated radiocarbon years before present (kyr cal BP) (Balme et al., 2018). Current evidence suggests dingoes did not reach the continental island of Tasmania, which was cut off from mainland Australia by post-glacial sea level rises around 11 ka; it is therefore broadly accepted that dingoes were introduced to Australia after this point in time (Corbett, 1995). Prior to the introduction of the dingo there had been no canids in Australia. Following their human-mediated translocation, dingoes spread rapidly throughout the mainland, inhabiting the full spectrum of habitat types from deserts to rainforests to the alpine highlands of the southeast (Purcell, 2010:20).

Dingoes are the largest extant terrestrial predators in Australia (Breckwoldt, 1988; Corbett, 1995; Purcell, 2010; Smith, 2015a). They are medium-sized canids, with adults weighing between 9–21 kg (average, 16 kg; Purcell, 2010). Dingoes live communally and hunt alone or in packs, and their social organization and reproductive processes, including parental behavior, are comparable to those of wolves (e.g., one annual oestrus cycle; Breckwoldt, 1988; Corbett, 1995; Purcell, 2010; Smith, 2015a). Females reach breeding age at about 12–24 months (Catling et al., 1992). As with wolves, and most wild canids, dingoes use underground dens to birth and nurse their young (Breckwoldt, 1988; Smith and Vague, 2016; Hudson et al., 2019). Dingo pups are reliant on intensive biparental care up until the age of 16 weeks (Thomson, 1992). Weaning usually begins about 3 weeks after birth (Smith, 2015a:36).

Dingoes are a distinct group of wild-living canids that together with the New Guinea singing dogs (Koler-Matznick et al., 2007) are genetically related to modern and ancient East Eurasian dogs (i.e., appear to descend from the ancestral wolf population that gave rise to domestic dogs; Bergström et al., 2020; Gojobori et al., 2021; Perri et al., 2021; Bergström et al., 2022). Hence, some may question the applicability of non-wolf analogies for our understanding of the human-wolf relationship in Late Pleistocene Eurasia. But dingoes behave as wild canids; from both behavioral and evolutionary standpoints, *C. dingo* is regarded as being intermediate between wolves and dogs (Smith, 2015a; Shipman, 2020; Koungoulos, 2021; Shipman, 2021). The human-dingo model can therefore provide insight into the possible nature of the interspecies companionship that arose between foragers and wolves in Late Pleistocene Eurasia and both preceded and led to the domestication of the latter (see also Clutton-Brock, 1995; Shipman, 2020; Koungoulos, 2021; Shipman, 2021; Brumm and Koungoulos, 2022), as has long been conjectured (e.g., Jones, 1970).

## Aboriginal peoples’ relations with dingoes

At the time of the European colonization of Australia in 1788 most Aboriginal people lived in small foraging communities that moved seasonally between resource areas within a defined territorial range, although population density and patterns of mobility and settlement varied across the continent according to rainfall and other environmental features (Keen, 2004). For example, compared with desert groups in the interior, coastal groups that were highly dependent on marine and estuarine resources were up to 100 times larger (in terms of estimated population densities), occupied a much smaller residential area, and moved their home bases far less frequently (Keen, 2004:125). During the early colonial period, Europeans commonly observed Aboriginal people cohabitating with dingoes (Phillip, 1789:174–175; Tench, 1789; Hunter, 1793:67; Collins, 1798: 567; Dawson, 1830:176; Nind, 1831; Mitchell, 1839:347; Grey, 1841:279; Morgan, 1852:39; Smyth, 1878:146; Dawson, 1881:89; Curr, 1886:47; Beveridge, 1889:112–113; Giles, 1889:20). The oldest records date to the last decades of the 18th century. In a few remote regions, 20th century anthropologists were able to observe firsthand what seem to have been unhybridized dingoes living in Aboriginal communities that had only recently had contact with Europeans (Thomson, 1957; Gould, 1969, 1970; Tindale, 1974; Gould, 1980; Tonkinson, 1991).

### The roles of dingo pups in aboriginal communities

Historical sources suggest wild dingoes were a valued food source in some but not all areas, and thus widely hunted, apparently by stalking and spearing adults (and sometimes ambushing them at waterholes), and stealing pups from dens (Breckwoldt, 1988). The pups (especially very young, fat ones) were a succulent delicacy, and highly sought after by Aboriginal hunters (e.g., Nind, 1831:29; Morgan, 1852:39; Giles, 1889:20; Tindale, 1974:109). Some groups, however, found dingo flesh unpalatable and hence regarded the animals as a low-ranked prey item (Meggitt, 1965). Others abhorred and/or prohibited the practice of consuming dingo meat (Kolig, 1978).

Numerous observers recorded that some dingo pups were kept alive specifically with the intention of raising them in human society (Meggitt, 1965). These so-called “camp dingoes” had varied roles in Indigenous communities (Meggitt, 1965; Gould, 1969, 1970; Jones, 1970; Hamilton, 1972; White, 1972; Kolig, 1973; Macintosh, 1974; Hayden, 1975; Macintosh, 1975; Barker and Macintosh, 1979; Gould, 1980; Gollan, 1982, 1984; Breckwoldt, 1988; Rose, 1992; Corbett, 1995; Meehan et al., 1999; Smith and Litchfield, 2009; Cahir and Clark, 2013; Smith, 2015b; Balme and O’Connor, 2016; Koungoulos, 2017; Shipman, 2020; Koungoulos and Fillios, 2020a,b; Brumm, 2021; Koungoulos, 2021, 2022; Shipman, 2021; Brumm and Koungoulos, 2022). Some early writers commented on the practical value these animals had as hunting aides (e.g., Dawson, 1881; Giles, 1889:20), although there is continuing debate on this subject (Balme and O’Connor, 2016; Koungoulos and Fillios, 2020a; Koungoulos and Fillios, 2020b). It was also recorded that dingoes served as sentries; protecting the community from malevolent spiritual forces as much as from human intruders (Kolig, 1973; Gould, 1980:23; Tonkinson, 1991:48; Meehan et al., 1999:93). They were also prized by some Aboriginal people as bed warmers (“living blankets”; Meggitt, 1965; Tindale, 1983/2005), and simply for their companionship (Basedow, 1925:118).

### Raiding dens for dingo pups

Most authorities agree that Aboriginal people did not intentionally control the breeding of camp dingoes (Smith and Litchfield, 2009; Shipman, 2020; but see below). Instead, the available accounts consistently state that they acquired dingo pups by conducting raids on wild dens (Dawson, 1830:176; Nind, 1831). Specific details about the den raids are lacking in most early accounts (Brumm, 2021). Kimber (1976:143) notes, however, that during the dingo whelping season in central Australia, ‘Walpiri and Pintubi men were energetic in their attempts to catch wild dingo pups’. At least in some areas, dingo den-raiding was a pre-planned, community-wide endevour. For example, the Pitjantjatjara people of central Australia organized special-purpose expeditions during the midwinter pup whelping season to raid wild dingo dens, with the intention of obtaining newborn pups as food and pets (Tindale, 1983/2005). This seasonal activity was linked to closely observed astronomical cues and deeply embedded in ceremonial life. Tindale recorded that the rise of the Pleiades open star cluster (the Seven Sisters) prompted the performance of a cycle of dingo increase ceremonies — sacred rites performed in order to promote the species’ continued fertility and abundance (see also Rose, 2012:56). Male initiation rites were also performed. Following these ceremonial activities, people would split into ‘small family and larger clan-like groups’ (Tindale, 1983/2005:374), each with recognized territories in which they were permitted to harvest dingo pups from wild dens.

Pups were usually taken from dens at a very young age, when they were pre-weaned, largely immobile, and completely dependent on parental care. As Smith comments:

[Aboriginal people] preferred the dingoes as young as possible, usually around two to four weeks old. This suggests an understanding of the critical period of socialisation in canids, where there is only a small window of opportunity to socialise a pup to humans in order for it to be a successful and well-adjusted pet later in life (Smith, 2015b:87).

Some evidence suggests that, upon raiding a den, the choice between which individuals within the litter of newborn pups were killed for food, and which would be kept alive to rear as companions in camp, was not necessarily an arbitrary one. For example, Pitjantjatjara people would usually kill most of the pups in the den for food, ‘but some particularly marked and semi-albinic animals may be kept’ (Tindale, 1983/2005:374). Similar practices were recorded among another inland desert people, the Ngaanyatjarra: ‘Normal wild [dingoes] are light brown in color but aborigines tend to select odd-colored variants from wild litters on which they feed’ (Tindale, 1974:Plate 79). In addition, Warlpiri people killed and ate weak or deformed pups (both male and female) they found, but they ‘kept unharmed the sound pups’ (Meggitt, 1965:14). Ethnographic evidence therefore seems to suggest that the den-raiding and pup-adoption practices of Aboriginal people involved a process of selection on morphology and other characteristics from the outset (Smith and Litchfield, 2009).

### Rearing pups in camp

Pre-weaned pups that were taken from dens and selected to live with people as social companions were hand-reared with loving care and affection. Women most commonly undertook this surrogate “mothering” role (Nind, 1831:29; Mitchell, 1839:347). Favored pups were subjected to an intensive process of nurturing and socialization that involved: breast-feeding of pre-weaned pups (Mitchell, 1839:347; Grey, 1841:279; Hamlyn-Harris, 1918:5); continuous ‘petting and pampering’ (Basedow, 1925:118); grooming pups for parasites (Hamilton, 1972:288); and carrying them around in the same position as (and commonly alongside) human babies (Hamilton, 1972:288).

Reports describe the strong emotional attachments between camp dingoes and their adoptive parents or carers (Balme and O’Connor, 2016). Unlike other animals kept as pets (Philip and Garden, 2016), camp dingoes were given personal names and kin classifications that placed them within a web of social relations with humans (Thomson, 1957:18; Rose, 1992:176). They also lived and slept together with their carers and wider human family, and were reputedly ‘indulged’ (that is, played with and coddled incessantly) as though they were small children (e.g., Krefft, 1865:372; Mathew, 1910:79). When the human-reared dingoes died, their human family mourned their loss with profound grief (Curr, 1886:47). Dingo burials (both with and without people) are known from the archeological record (Gunn et al., 2010; Koungoulos and Fillios, 2020b), although they are poorly studied (Koungoulos, 2021). It is also known that dingoes have prominent and varied roles in the cosmologies of many Aboriginal groups, including as mythological figures (Kolig, 1973; Rose, 1992; McIntosh, 1999).

Despite all this, historical accounts and anthropological observations suggest a consistent cultural pattern in which very young pups were intensely nurtured and doted upon, well-fed and so on, but as they grew out of the cute puppyhood phase — and thus presumably became less appealing as objects of human affection — they were largely left to fend for themselves (Meggitt, 1965; Meehan et al., 1999). These dingoes appear to have still been physically and sexually immature, but they were no longer actively provisioned by humans and hence had to survive by scavenging and scrounging discarded food remains. European observers commonly portrayed these dingoes as appearing to be severely emaciated (e.g., Mitchell, 1839:102–103). For example, at one desert camp Gould (1970:65) described an elderly woman’s coterie of pet dingoes as ‘the skinniest dogs I have ever seen’ (Gould, 1970:65). Early written accounts suggest that the hungry young dingoes often left camp to hunt for themselves, but were so closely bonded to humans that they would return to camp after a few days (Nind, 1831:29). Notably, most observations of this sort were made in the arid zones of Australia’s interior, especially deserts, where food is scarce. It is unclear if the same pattern was at play in more resource-rich parts of the country (Meggitt, 1965:17).

### Status of mature camp dingoes

The early writers are largely silent on this issue of what happened to the young socialized companion dingoes when they were physically mature. To our knowledge, there are few confirmed observations of breeding-age females or adult males consistently being present in Aboriginal camps (Brumm, 2021; Koungoulos, 2021, 2022). It should be noted that there is little evidence to suggest that Aboriginal people slaughtered camp-living dingoes with the intention of consuming them, except during times of extreme hardship — and even then with great reluctance (e.g., Gould, 1980:241). Dingo body parts (skins, teeth, bones, and so on) were observed in use as material culture items (e.g., dingo tail headbands) in various parts of Australia (for detailed reviews of such uses see Philip, 2016 and Koungoulos, 2021). However, there are no indications either from available ethnographic accounts or the archeological record that Aboriginal people consistently used the remains of human-reared dingoes as culturally modified objects — which might have provided a motivation for harvesting pups raised in captivity (e.g., Germonpré et al., 2018). In cold areas, warm garments like cloaks and rugs were fashioned from possum skins (Wright, 1979). In modern Aboriginal communities there are often prohibitions against killing dogs: ‘To harm one, even by accident, is a serious offense punishable by a fine of hundreds of dollars and a public beating’ (McIntosh, 1999:188). According to Gould (1980:246), ‘there is every reason to think that this was true for dingoes in pre-contact times as well’.

The most commonly held notion among present-day scholars — based principally on the testimony of Aboriginal informants — is that when human-raised dingoes reached reproductive age (~12–24 months) they reverted permanently to the wild to breed (Meggitt, 1965; see also Lumholtz, 1889:196; Thomson, 1957:16; Hamilton, 1972:288; Meehan et al., 1999:92–93). The question remains: if the tamed and socialized camp dingoes had access to a regular source of human-derived food as they got older, would they have chosen to remain with their adoptive human family? There is evidence to suggest that some Aboriginal groups may not necessarily have favored such an outcome. Certain communities had a cultural prohibition against mature dingoes residing in camp because the reproductive behavior of these canids (e.g., incestuous coupling) diverged so dramatically from that of humans (Kolig, 1978; Tonkinson, 1991:22; Rose, 1992). Camp dingoes that did not revert to the wild when they reached breeding age may have been ousted or killed for this reason. According to the traditional exegesis of the Yarralin (northern Australia), dingoes who would not leave when they became sexually active ‘were destroyed by a poison derived from an orchid’ (Rose, 1992:176).

### Dingo management in pre-contact southeastern Australia

**Figure 2:**
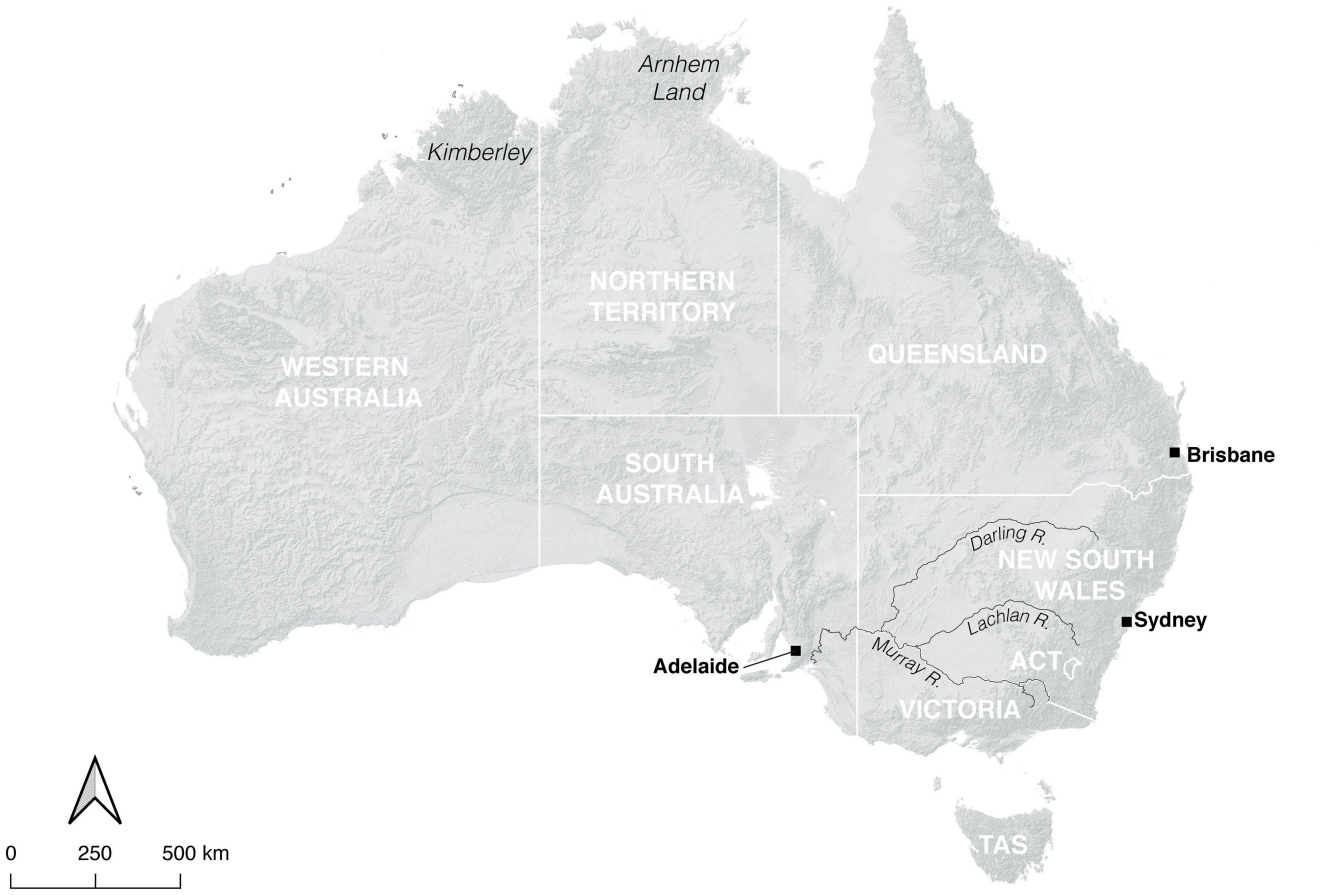
Map of Australia. The major rivers of the southern extent of the Murray-Darling basin are shown. Base map: Kim Newman.

Exceptions to the above pattern may have been found in recent pre-colonial southeastern Australia, broadly defined as the area approximately between Adelaide and Sydney, bounded on the west by the limits of the Murray-Darling river system and in the south and east by the coastline (Figure 2). Within this region, deceased tame dingoes were frequently buried *via* inhumation in the same manner, and often alongside, Aboriginal people (Pardoe, 1996), constituting a tradition apparently unique to this part of the Australian continent and communicating the importance of tame dingoes to local communities. An osteometric study of a small subset of these individuals identified that some of them had reached or were long past the onset of sexual maturity, and that some displayed morphological alterations from the (otherwise highly conservative) wild morphotype, suggestive of isolated reproductive pools centered around Aboriginal settlements (Gollan, 1982). These changes included reductions in the gross size of dentition, and a pronounced gracility of size and shape in cranial proportions; most notably shortened and broadened rostrum, steeper face and brow, rounded and shortened calvarium, reduced sagittal and nuchal cresting. Together these traits are reminiscent of domestication-related morphological changes noted between dogs and wolves (Drake et al., 2015; Geiger et al., 2017; Galeta et al., 2021).

The question of whether such individuals were *domesticated* depends on the definition employed. In the biological sense focusing on presence of phenotype changes, the evidence is equivocal. The reproduction of tame dingoes within the camp is entirely plausible given the long-term residences of individuals for years into reproductive maturity and the occasional presence of puppy remains, but there is no evidence that there was any degree of human influence over this process. The morphological changes ostensibly resulting from domestication-related selection are variable, and their origin poorly understood. The clearest cases of dental reduction and altered cranial proportions seemingly did not occur within the same individuals, and potentially were driven by unrelated processes that could reflect epigenetic or early developmental factors rather than impacts of long-term selection (Koungoulos, 2021). They may potentially also relate to deep-rooted genetic divergences that are unrelated to human influence and predate these specimens by thousands of years (Koungoulos, 2021). Finally, most specimens assessed by Gollan (1982) showed no phenotypic difference to wild dingoes, despite signs of anthropogenic influences on their diet, indicating they were most likely born to wild parents lacking a history of anthropogenic selection. This suggests that in the long-term camp-dwelling dingo populations could not be sustained by internal reproduction and required replenishment from wild dens, probably owing to high juvenile mortality and adult dispersal.

Conversely, envisioning domestication as a social or cultural process based on enduring relationships between animals and people (Bogaard et al., 2021; Losey, 2022), it would seem that tame dingoes which did not depart the camp but willingly accompanied people for the duration of their lives were not functionally distinct from the undifferentiated free-ranging domestic dogs found in many other Asia-Pacific societies. Although notable differences in disposition, intelligence, and independence probably existed between these dingoes and modern domestic dogs, these individuals had successfully become adapted to life alongside people in a domestic environment. This is particularly notable because this seems to have also been achieved without the use of physical confinements – an enormous challenge for modern dingo-keepers (Brumm and Koungoulos, 2022). Although specific evolutionary or physiological mechanisms generating changes in the morphology of some tamed dingoes are presently not understood, they are not observed in wholly-wild dingo populations and should be regarded as the eventual result of inter-generational dingoes reproducing within camps, or the sourcing of wild-born pups from parents who had previously been raised in camps.

Gollan (1982) speculated that aspects of the mobility and food-procurement pathways characteristic of Aboriginal societies in southeastern Australia were responsible for facilitating the long-term retention of dingoes within or around Aboriginal camps. Koungoulos (2022) has expanded upon this notion, arguing that a particular confluence of social and economic factors was conducive to the long-term management of tame dingoes and the emergence of altered phenotypes among them. Southeastern Australia was home to the largest, densest, and least mobile Aboriginal communities in the continent (Clarke, 2009; Pate and Owen, 2014; Pardoe and Hutton, 2020). The demography and lifestyles of these communities were supported by a well-watered and productive landscape offering abundances of aquatic animals and carbohydrate-rich wetland plants, and in the lower reaches of rivers estuarine and marine resources could also be accessed (Clarke, 2002; Pate, 2017).

Abundance of fish in particular offered a reliable resource base which could be used to provision large and long-term populations of camp dingoes as an alternative to sacrificing terrestrial dietary protein (and the valuable fats of mammalian and avian bone marrow) from the diets of people (Koungoulos, 2022). Two dingo burials studied by Gollan (1982) indeed had marine fish remains, presumably originating *via* human fishing, preserved in their stomachs. Marine and aquatic resources are frequently identified as important food bases for the management of prehistoric dogs in contexts outside Australia (Clark, 1997; Gonzalez et al., 2013; Losey et al., 2013; Perri et al., 2019). The general abundance of faunal resources in these environments may have also furnished an attractive base of scavengeable discard material within and around Aboriginal occupational sites that lessened the impetus for tame dingoes to stray far from the camp for self-foraged sustenance (particularly while raising pups).

Within such a landscape where larger, more closely-spaced and longer-resident Aboriginal communities were practicing the annual rearing of dingoes taken from dens, the relative probability of human influence acting inter-generationally upon these dingoes must have been greater. This could be because settlements were, through the availability of resources, more capable of sustaining several generations of tame dingoes reproducing within their confines if so desired. More interestingly, pups born to formerly tame dingoes which had left the camp to reproduce in the wild would also have had a greater chance of being subsequently collected from the den by members of the same community or a neighboring one for taming (Brumm, 2021) — by virtue of there being more settlements within close proximity to available denning locations, more people seeking to collect dingo pups, and their being more likely to do so within the same localities year-after-year (Koungoulos, 2022). If this same pattern was repeated over many generations, then a longstanding practice of raiding dens for pups and hand-rearing the favored ones may have modified the genetic makeup of the wider dingo population, even if the socialized canids typically dispersed into the wild when they were sexually mature (Brumm, 2021). Regardless of whether pre-contact southeastern tamed dingoes qualified as “domesticated,” their life-histories are of clear relevance to discussions regarding human-canid interactions potentially leading to domestication and the development of “dogs” which complete their life-cycles within a domestic setting.

## Implications of human-dingo relations for wolf domestication

The available insight into the human-dingo relationship in Australia conflicts with two key interrelated aspects of the human-initiated model of wolf domestication. Firstly, rather than keeping hand-reared pups within or close to human society when they reach sexual maturity — in order to acquire the next generation of pups — Aboriginal people typically allowed socialized dingoes of breeding age to revert to the wild, chased them away, or even killed them to prevent them from reproducing within the milieu of human domestic life. This presents a challenge for the scenario outlined by Mech and Janssens (2022) for the earliest beginnings of wolf domestication, in which the socialized adult wolves were permitted (even encouraged) by humans to breed with siblings and close relatives that were raised similarly in camp or with canids present in the camp that had been obtained as gifts or barter by other human groups (see also Germonpré et al., 2021a). Secondly, Aboriginal interactions with dingoes challenge the notion that acquiring new wolf pup companions by stealing them from wild dens was largely confined to the very earliest phases of the human-wolf relationship that gave rise to the ancestors of modern domestic dogs.

The latter point is noteworthy. As has been previously mentioned, the pup-keeping hypothesis of wolf domestication proposes that Late Pleistocene hunter-gatherers took the very first generations of pre-weaned wolf pups from the natal dens of free-ranging wolves and reared them in camp; the sexually mature wolves then began to breed in human captivity, giving birth to pups within the confines of foraging settlements. There is therefore a tacit assumption that only a few generations after the first pups were brought in from the wild to live in human communities it would no longer have been necessary to raid wild dens to acquire these companion animals. Nevertheless, wolf domestication would have involved a long and anastomosing pattern, and the process should not be equated to a simple path ending with the emergence of the first modern dogs (see Germonpré et al., 2012, 2021a; Galeta et al., 2022; Losey, 2022).

With regards to the case in Australia, as Meggitt (1965:22) points out, ‘the very generality and persistence of the practice of taking dingo pups alive suggest that, for whatever reasons, the numbers in the camps had constantly to be replenished’. Meggitt (1965) infers that some camp dingoes may well have remained in camp as adults; unlike domestic dogs, however, the breeding habits of captive wild dingoes may have been disrupted by the presence of humans. Hence, the socialized camp-living dingoes may not have produced new pups ‘at a rate high enough to counterbalance the constant loss of tame dingoes through death or running away’ (Meggitt, 1965:23).

Even if they did, however, the practice of raiding dens may have continued for reasons that are related more to the roles of dingoes in human thought and cultural belief than pragmatic considerations. For example, it seems noteworthy that Yolngu people in Arnhem Land still raid bush dens to obtain the pups of wild-living canids, which they consider to be dingoes and are raised as pets in their home communities (N. Fijn pers. comm., 2021). This practice endures despite the fact that Indigenous people in these remote settlements have long possessed European domestic canines (“camp dogs”) that reside with them permanently (Maher et al., 2019). Moreover, the wild-living canids found in these dens, which are reasonably close to Yolngu settlements, now probably consist predominately of free-ranging dogs with some dingo ancestry, rather than genetically pure *C. dingo* (N. Fijn pers. comm., 2021; see, e.g., Bombara et al., 2017); although it is likely that these hybrids fill the same ecological niche as pure dingoes and exhibit broadly the same behavior. Yolngu value the independent characteristics of the dingo (especially its hunting prowess) and the significant totemic connections of these canids among certain clans; they also believe that it is important for various animals to be allowed to be ‘wild’ (N. Fijn pers. comm., 2021). It is no longer necessary to raid bush dens in order to obtain newborn canids for use as companions; however, the practice of den-raiding continues because it is a part of the ongoing expression of a perceived spiritual connection between Yolngu people and free-ranging dingoes (e.g., Rose, 1992).

In sum, the human-dingo model would appear to subvert two of the main assumptions of the pup-keeping hypothesis of wolf domestication. A key question therefore remains: if the human-wolf relationship in Upper Paleolithic western Eurasia was similar to or at least compatible in nature with the human-dingo relationship in Australia, would it still be possible for domestication to occur?

**Figure 3:**
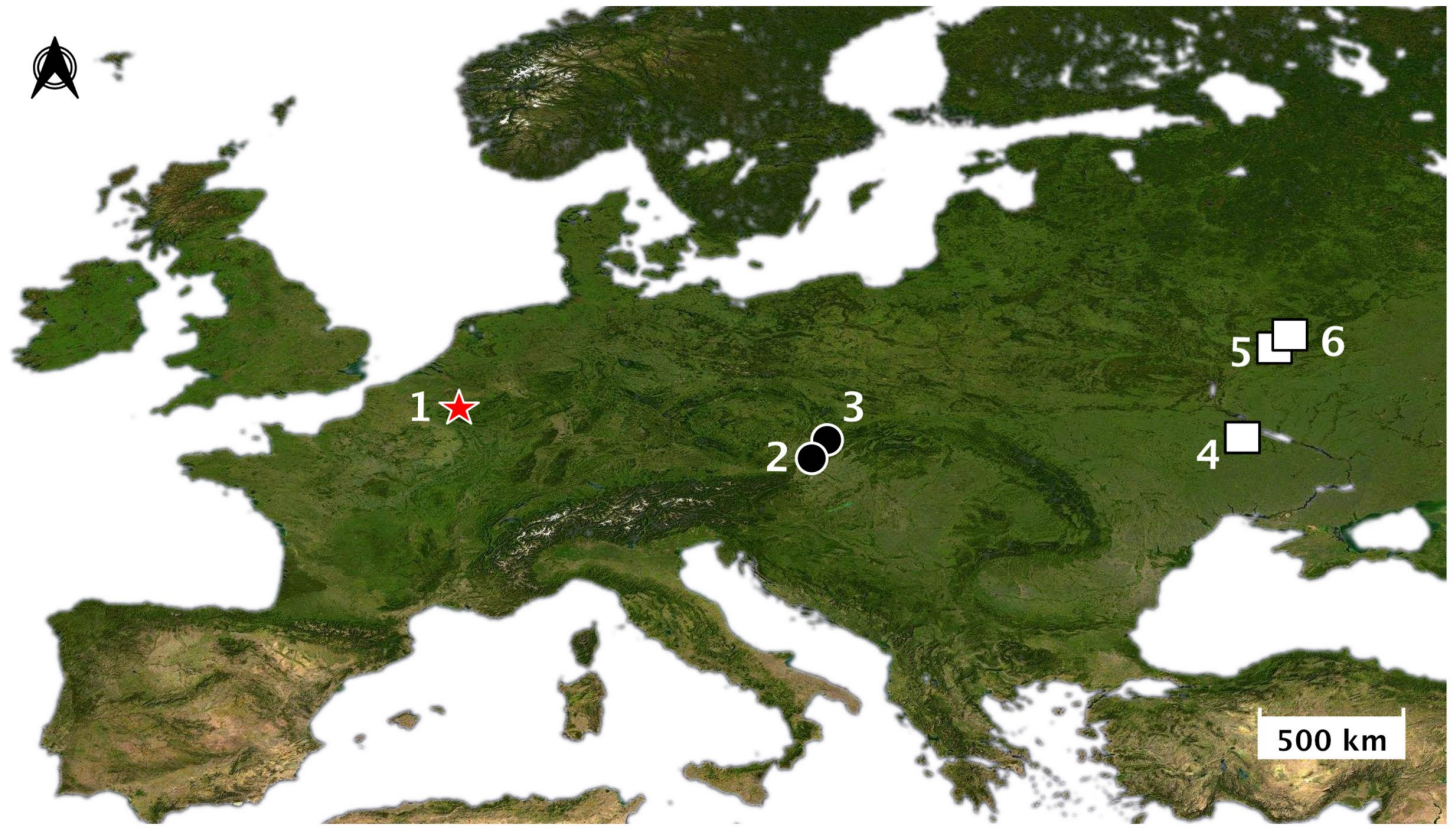
Map of Europe showing the location of sites mentioned: (1) Goyet; (2) Předmostí; (3) Dolní Věstonice; (4) Mezhirich; (5) Yudinovo; (6) Eliseevichi. Red star = Aurignacian sites; black circle = Gravettian sites; white square = Epigravettian sites. Base map: Europe-NASA-satellite. Credit: Elodie-Laure Jimenez.

**Figure 4:**
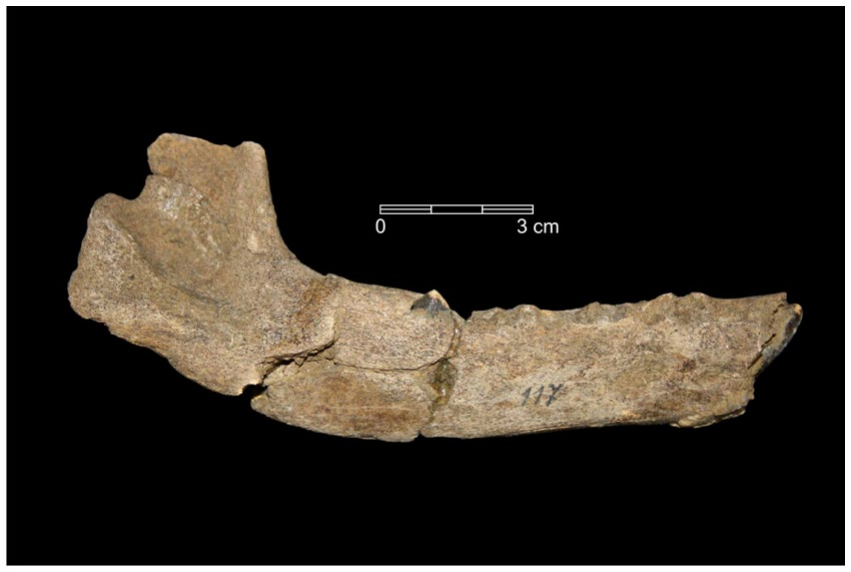
A right mandible from a large canid pup found at the Gravettian site of Předmostí (Czech Republic) that died when it was 4–5 months old (Germonpré et al., 2021a). The specimen is housed in the Moravian Museum, Brno (image credit: M. Germonpré).

The argument for an early and widespread practice of wolf pup adoption in Late Pleistocene Eurasia is broadly consistent with the archeological record — or at least is not directly refuted by it. A detailed study of the age distribution of the large canids found at Upper Paleolithic sites in Europe has not yet been undertaken. However, at the site of Předmostí (28.5 ka) in the Czech Republic (Figure 3), most canid skulls are clearly “adult” (~4–6 yo); for example, the dentition of skull Předmostí 3 has a wear stage corresponding to an age of 6–8 years old (Germonpré et al., 2012: table 3, Figure 4). There are a few remains of pups that were about 4–5 months old at time of death (Germonpré et al., 2021a; Figure 4). Modern female wolves can breed when they are as young as 10 months, but they generally reach first oestrus when they are 2–4 years old (Heptner et al., 1998; Mech and Janssens, 2022). In a study of Scandinavian wolves, median age at first reproduction was three years for females and two years for males, with a range of between one and 8–10 years (Wikenros et al., 2021). Over half (~52–60%) of the individuals (male and female) in this Scandinavian study population reproduced for the first time at 1–2 years of age.

Under the human-initiated pet-keeping model, it can be anticipated that many of the large canids living as companions at any given time in Late Pleistocene hunter-gatherer communities prior to when the first widely accepted domesticated dogs appear (~17 ka) would have been very young pups and juveniles (or adults) of non-breeding age. However, the presence of skeletal remains of adult canids at Upper Paleolithic archeological sites, or the absence or rarity of juveniles, does not refute this prediction. Concerning the latter, the remains of very young wolf pups are less likely to be preserved in the Late Pleistocene archeological record from a taphonomic standpoint — just as the skeletal remains of babies and very small children rarely survived at these sites.

**Figure 5:**
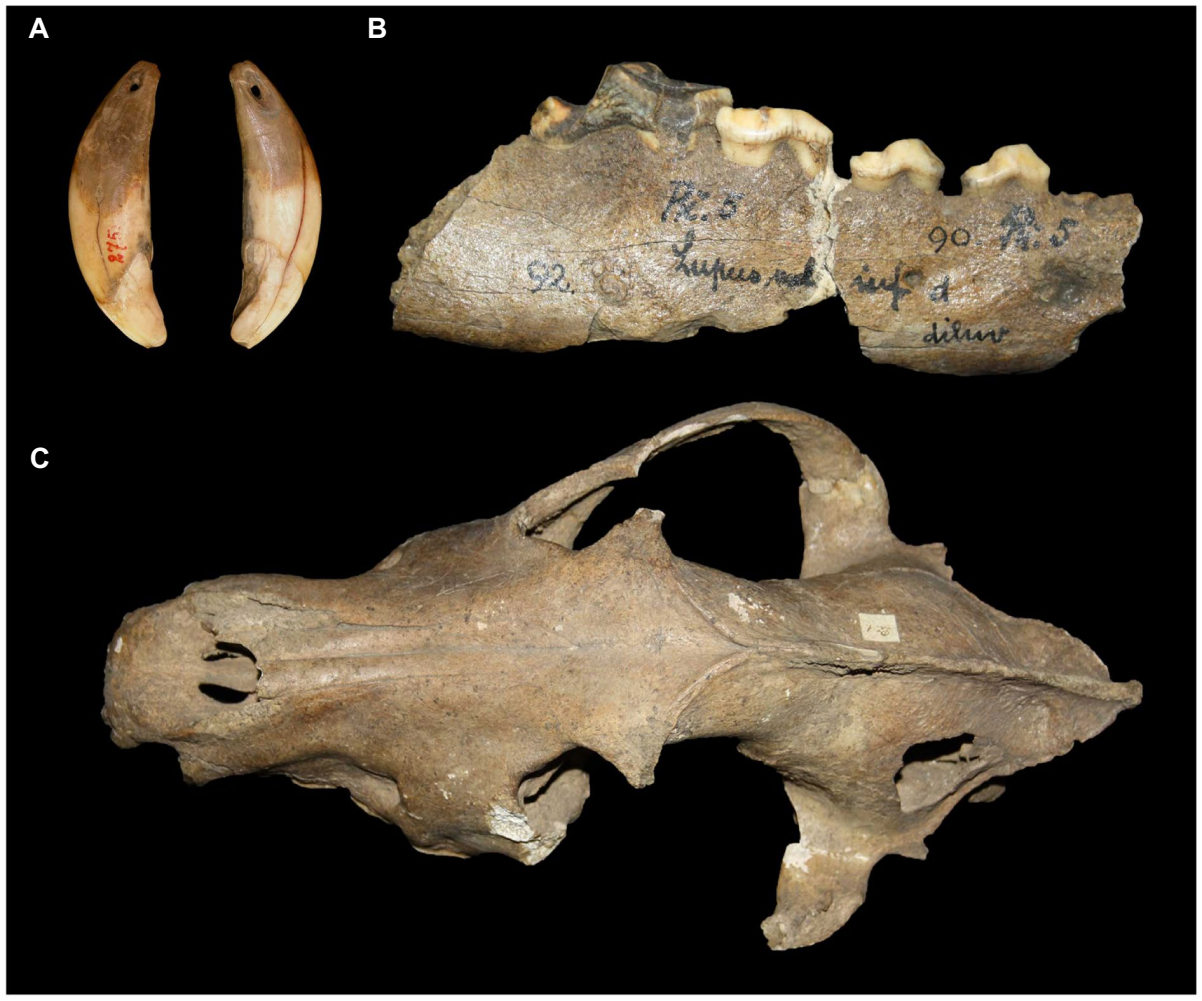
Images of Upper Paleolithic wolf remains with human modification. **(A)** Perforated canine from a large canid, Upper Paleolithic, Goyet cave, Belgium (Royal Belgian Institute of Natural Sciences [RBINS]; image credit: RBINS). **(B)** Impact marks on the ventral edge of a mandible from a Pleistocene wolf from the Gravettian Předmostí site, Czech Republic (Moravian Museum, Brno; image credit: M. Germonpré); crown length m1: 30.64 mm. **(C)** Dorsal view of a skull from a Pleistocene wolf from the Gravettian Předmostí site, Czech Republic, with a perforation in the left braincase (Moravian Museum, Brno; image credit: M. Germonpré); total skull length: 276.54 mm.

We would also expect to find adult wolf remains in human settlements, given the argument that some human-socialized pups would have lived with people as adults. Moreover, excavated archeological findings indicate that Upper Paleolithic people killed adult wolves for food and raw materials (e.g., meat, skins and fur; Germonpré et al., 2017a, 2018, 2021a). From the beginning of the Upper Paleolithic record there is an increase in the frequency of carnivore remains, including wolf remains, many bearing traces of human modifications (Collard et al., 2016; Figure 5). This could relate to the use of the bones and dentition of these animals as a resource for raw material for personal ornaments and tools, of their skins and fur to tailor cold weather clothing, for their meat for consumption, and their body or body parts to have a symbolic/ritual role (Germonpré et al., 2018, 2021a). We should therefore expect to find skeletal remains of mature canids with cut-marks and other evidence for processing at Late Pleistocene human occupation sites throughout Eurasia, as indeed is the case (Germonpré et al., 2018).

### Wider human interactions with socialized wolves

A vital component of the early human-wolf association based on capturing and raising wild pups is the nature of the relationship between humans and the socialized wolves once they attained breeding age. We contend that when a wild-caught socialized wolf reached sexual maturity it generally would have had a strong natural tendency to revert to the wild to breed. This is in keeping with our knowledge of modern wolf social ecology, wherein a young wolf of breeding age typically disperses from its natal pack to find a mate (Mech and Boitani, 2003). However, wolf behavior is flexible enough (da Silva Vasconcellos et al., 2012; Packard, 2012) that some individuals would probably not disperse if they could find appropriate sexual partners in the camp sites. The socialized wolves that were leaving Upper Paleolithic communities when they matured sexually were likely then in a natural life-history transition that would have been difficult for humans to circumvent. As noted, this behavior appears to have been commonplace among camp dingoes in Australia: indeed, some Aboriginal groups seem to have accepted that it was the “dingo way” to follow “the call of the wild” (i.e., revert to the bush to reproduce), and did not attempt to prevent the animals from departing. In some cases Indigenous foragers may even have actively encouraged hand-reared canids to return to the wild, ostensibly because the sexual behavior of dingoes transgressed the laws of human morality (Rose, 1992).

We agree with Mech and Janssens’s (2022) argument that socialized adult wolves that escaped or were released from camp, and which established their territory in areas with easy access to human occupation areas or kill sites, were likely to have denned and whelped in proximity to humans. Unsocialized wolves are unlikely to have displayed this behavior owing to their natural fear of humans (Sazatornil et al., 2016:108). If socialized adult wolves that reverted to the wild lacked the propensity to segregate their breeding sites from humans then it is reasonable to assume that their dens would have been more easily accessible to humans than those of unsocialized wolves, as was conceivably the case with dingoes in Australia (Brumm, 2021; Koungoulos, 2021, 2022). Consequently, we can think of dens used by “re-wilded” socialized wolves in the vicinity of human settlement sites, which can be considered as anthropogenic habitat “islands” (*cf.* Spengler, 2022), as being in a *liminal* zone between the environment inhabited by strictly wild-living (unsocialized) wolves and the camp sites occupied by humans.

It follows that if camp-living and dispersing socialized wolves commonly formed mated pairs with each other, then any offspring they produced are likely to have inherited morphological and behavioral characteristics that had been under human selection in one or both parents. Hence, we surmise that a long-term pattern of hand-reared, socialized wolves breeding in liminal zones near human camps may have influenced the enrichment of genetic variants (i.e., caused genetic changes) in these populations.

**Figure 6:**
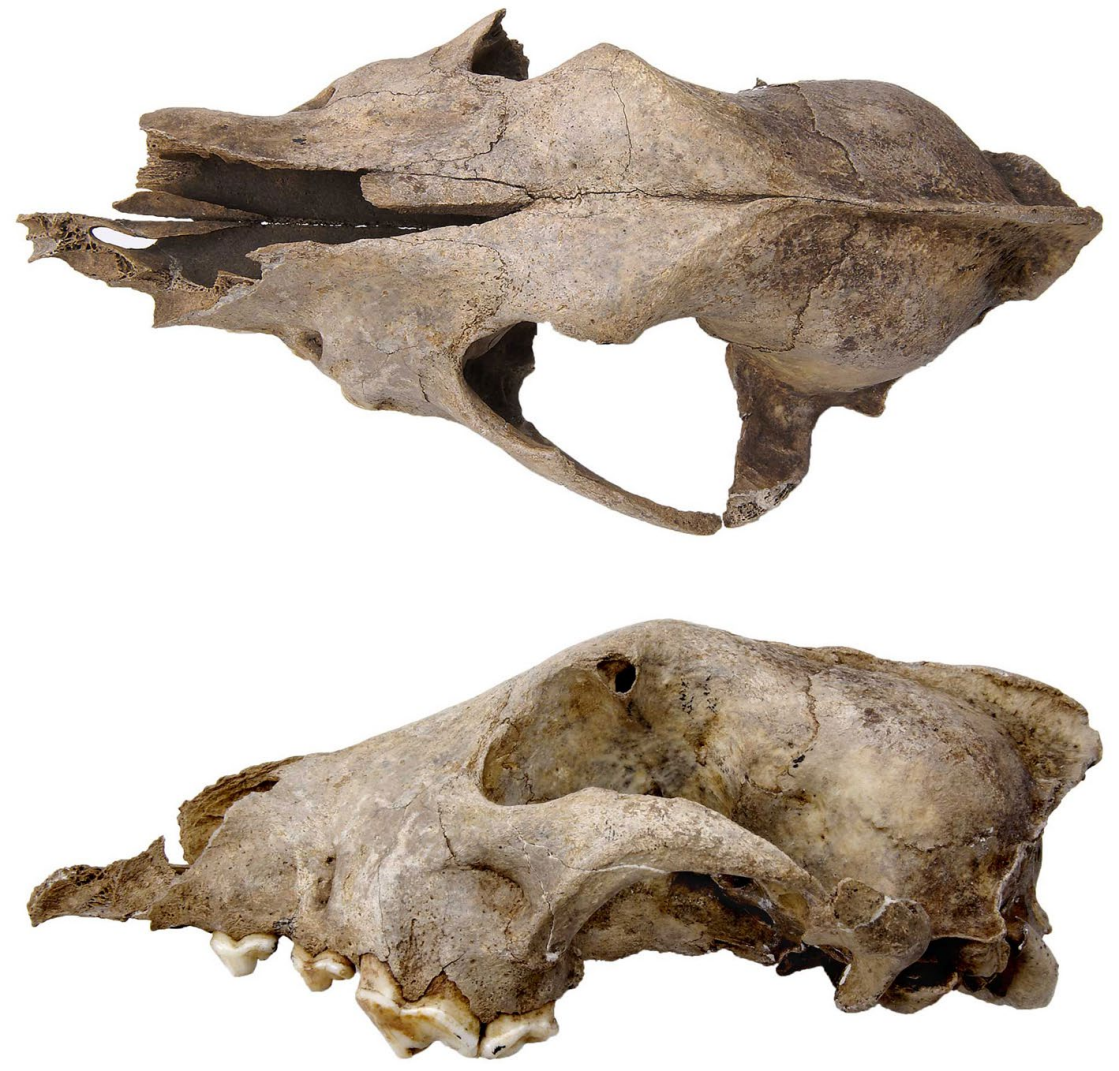
Dorsal (top) and lateral (bottom) view of the skull of the Paleolithic dog from Goyet cave, Belgium (RBINS; image credit: RBINS). Total skull length: 227 mm.

We would expect this process to have begun to produce recognizable changes in wild wolf populations long before the first widely accepted remains of domestic dogs appear in the archeological record after the Last Glacial Maximum (LGM). Evidence for this is provided by the early appearance of dog-like skeletal remains in western Eurasian contexts (Germonpré et al., 2009, 2012, 2015a, 2018, 2021a; Ovodov et al., 2011). In the faunal assemblages of several early and middle Upper Paleolithic sites, two sympatric canid morphotypes have been described. The Pleistocene wolf morphotype is rather similar in shape and size to recent northern wild wolves from Eurasia. The Paleolithic dog type (also referred to variously as ‘protodog’ or ‘Palaeodog’; Figure 6) has a smaller skull and a shortened snout with a proportionally wide palate, and a higher and shorter lower jaw compared to the Pleistocene wolf type (Germonpré et al., 2017b; Galeta et al., 2021, 2022). It has been suggested that these altered craniodental features of the Palaeodogs could have been caused by the inadequate diet they received as pups or that their mothers received while carrying them in their womb (*cf.* Zeuner, 1954; Platt and Stewart, 1968), by a restricted mobility as they were tethered or kept enclosed, by inbreeding and/or by selective breeding based on the sociable traits in some of the pups (Germonpré et al., 2021a). Koungoulos (2021) has suggested that the Palaeodog morphotype may actually include socialized adult wolves that had reverted to the wild but remained in a relationship of commensalism with humans, as has been proposed for dingoes (Brumm, 2021).

The oldest known specimen of a Palaeodog identified thus far is from Goyet Cave in Belgium (35.7 kyr cal BP; Germonpré et al., 2009; Figure 6). We infer that by this early stage in the modern human occupation of Europe the practice of adopting wolf pups from liminal dens had begun to alter the genetic makeup of some wild wolf populations, at least in this part of western Eurasia, but possibly more widely. We think this process of humans inadvertently changing wolves through their practice of taking their young from dens to rear as pets had become more advanced by the Gravettian (~33–24 ka). At this stage, a key shift in the human-wolf relationship came into effect when large groups of mobile hunter-gatherers began gathering at aggregation sites at times when wolves were denning and whelping nearby.

### Den-raiding near human aggregation sites

**Figure 7:**
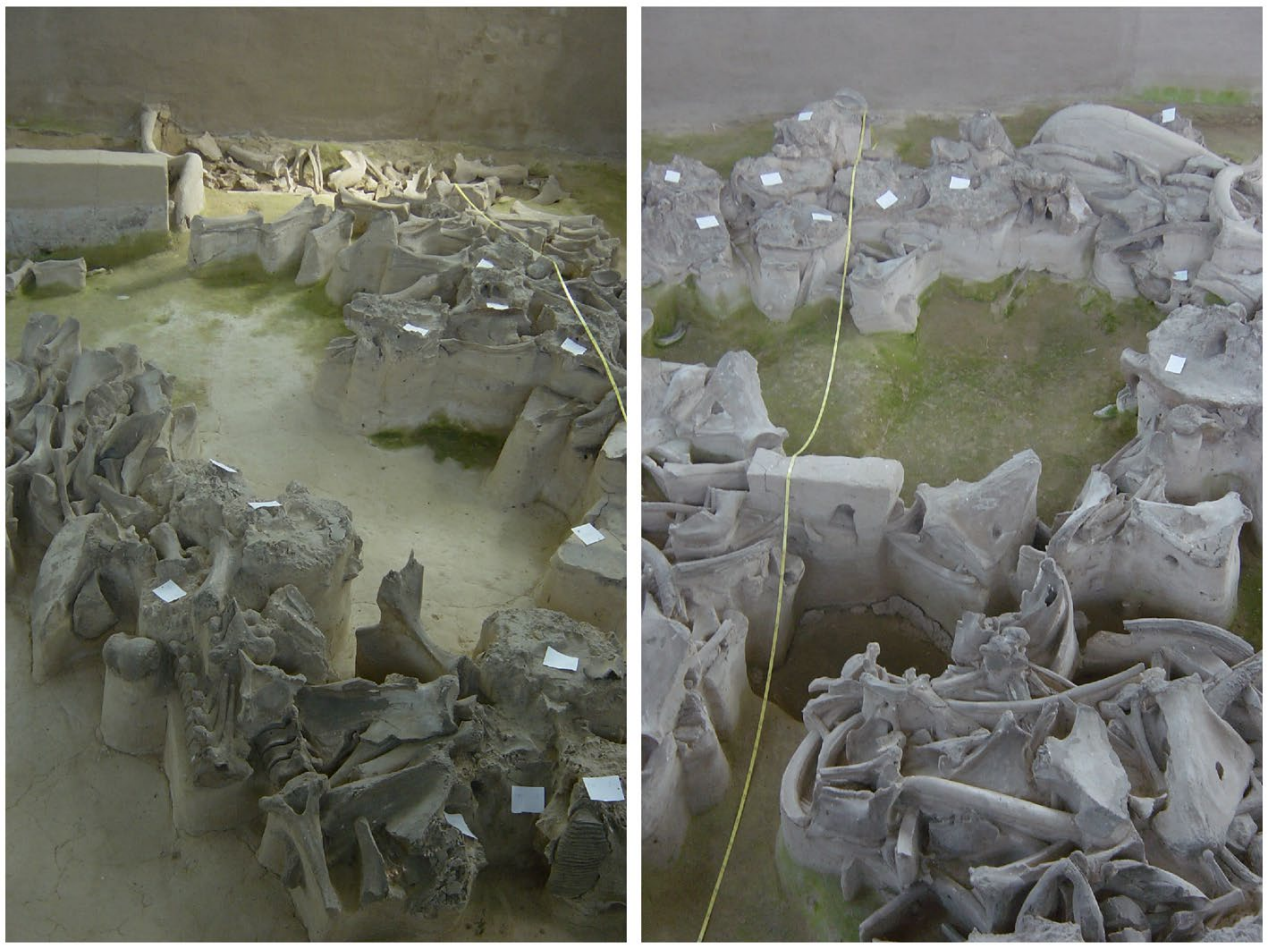
Upper Paleolithic mammoth bone complex. Left: Mammoth bone structure #4 at the Epigravettian Yudinovo site, Russia (image credit: M. Germonpré). Right: Mammoth bone structure #3 at the same site (image credit: M. Germonpré).

In this regard, we draw attention to the large open-air mammoth “megasites” in central and eastern Europe (e.g., Předmostí, Dolní Věstonice, Yudinovo, Eliseevichi, Mezhirich; Figure 7). Dating from the Gravettian and Epigravettian, these probably represent large aggregation sites (Soffer, 1985; Oliva, 1997; Pidoplichko, 1998; Germonpré et al., 2008; Khlopachëv and Polkovnikova, 2017; Khlopachev, 2019; Sablin et al., forthcoming). Many of these impressive sites are characterized by direct or indirect evidence for mammoth hunting, as well as human burials, female anthropomorphic figurines, procurement of exotic materials and/or substantial architectural constructions made from mammoth bones (Germonpré et al., 2020, 2021b). At these locations, a surplus of mammoth meat enabled large numbers of foragers to gather recurrently for an extended period of time (Sablin et al., forthcoming; Germonpré et al., 2021b). Based on seasonality studies, it can be inferred that people were often concentrated at these sites during several seasons, including spring (Nývltová Fišáková, 2013; Germonpré et al., 2021b), precisely the time of year when wolves would have been denning and whelping (Mech and Boitani, 2003).

These sites are also notable for the presence of both Pleistocene wolves and Palaeodogs. Furthermore, these two morphotypes represent ecologically distinct populations that differ in their dental microwear textures (Prassack et al., 2020, 2021) and stable isotope ratios (Bocherens et al., 2015), suggesting that the Pleistocene wolves ate more suitable and softer food items that contained a large portion of mammoth meat compared to the Palaeodogs. The latter had more bones in their diet, which consisted mostly of reindeer and muskox (Bocherens et al., 2015; Prassack et al., 2020, 2021). Likely, the Pleistocene wolves scavenged mammoth carcasses at human kill sites, like other carnivores also did, while Palaeodogs were fed lean meat (Bocherens et al., 2015; Lahtinen et al., 2021). The analysis of the anatomically modern human mandible recovered from Předmostí suggests that this person ate mainly mammoth meat and had as well a portion of wolf meat in their diet; they did not eat meat from Palaeodogs, however (Bocherens et al., 2015; Germonpré et al., 2017a,b). Reindeer were also hunted at Předmostí, as shown by their cut-marked remains (e.g., astragali; Germonpré, unpublished data). Their fur and marrow in addition to their meat would have been very useful for the Gravettian inhabitants of the site.

Importantly, therefore, in some parts of the Late Pleistocene Eurasian landmass (e.g., the Moravian Gate and Russian Plain), by the Gravettian and Epigravettian periods, there may have been a continuous cycle of (1) mobile groups of hunter-gatherers returning regularly to specific congregation sites (e.g., Předmostí), accompanied by young socialized canids which they had obtained previously from liminal dens located in the vicinity of these seasonal camps; (2) some of these socialized canids of breeding age dispersing into the wild (i.e., “re-wilding”) from these seasonal camps; and (3) hunter-gatherers congregated at these campsites replenishing their supply of wolf pups by raiding liminal dens, a practice that is likely to have involved revisiting known denning areas they had targeted previously [On Ellesmere Island, Mech (2022) observed free-ranging wolves reusing the same two dens to rear their pups every year between 1987 and 2006, with the exception of 1990 and 1991; one particular cave den on the island seems to have been reused ‘for centuries’ (Packard, 2003:37)].

**Figure 8:**
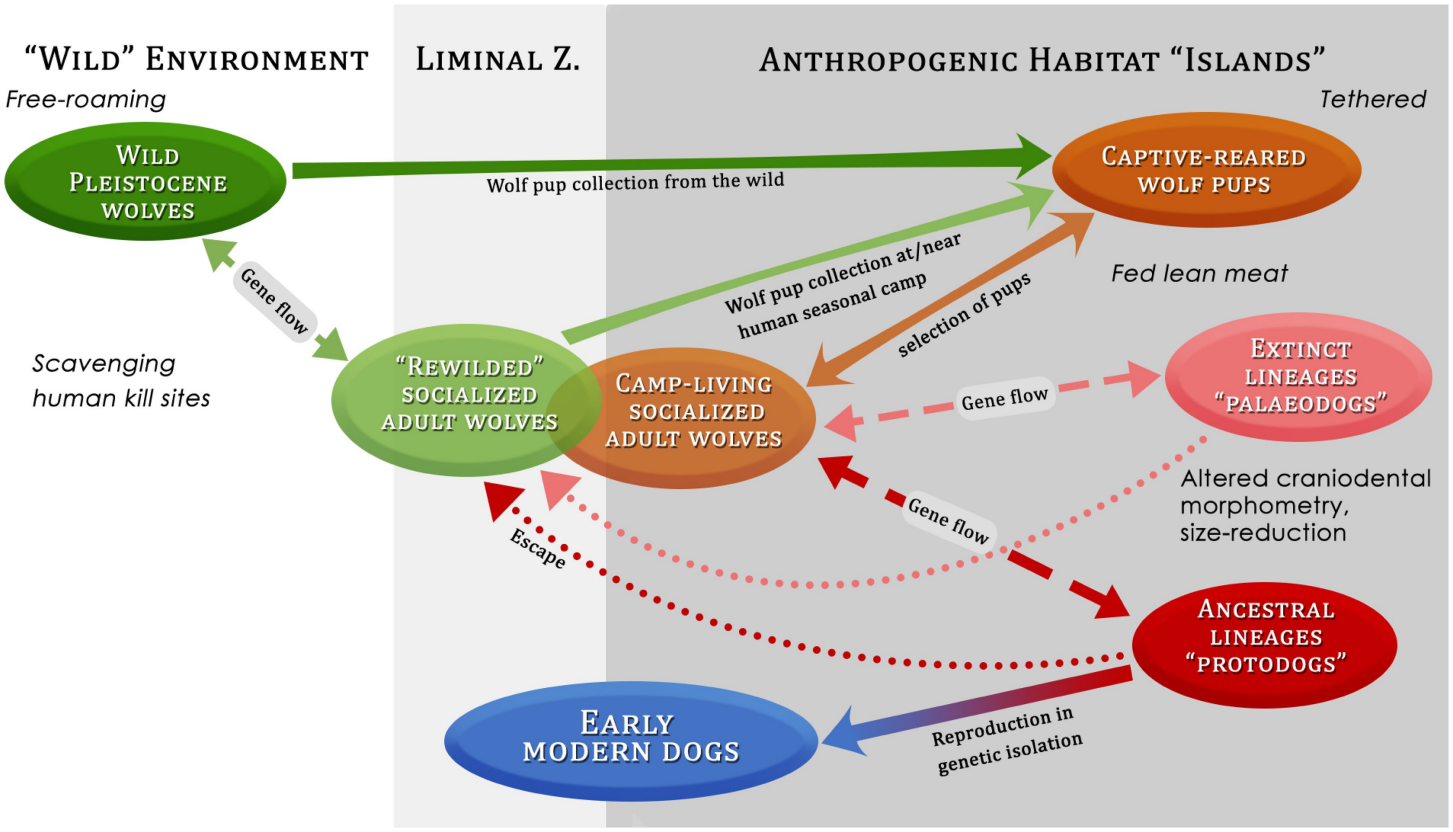
Possible pathways in the domestication process of dogs from wolves, showing several factors such as environment, selection, protection, food and provisioning. Liminal Z = Liminal zone.

Such a pattern, if repeated over a number of generations, could have had a pronounced effect on the population of canids from which humans routinely acquired new pups (Figure 8). First of all, following Mech and Janssens’s (2022) model, we can expect that newly “re-wilded” socialized canids commonly formed mated pairs with other dispersing socialized wolves and/or with former socialized wolves that had dispersed during prior seasons, and which had now congregated near the human seasonal camps or with the tamed canids kept at the camp sites. These human-reared canids are likely to have marked-out territories in the vicinity of human settlements, a key source of food, and prevented wild wolves from encroaching (Mech and Janssens, 2022). Women and children, in particular, but also men, may have been motivated by emotional ties to re-establish close bonds of companionship and communication with the canids they had raised as pups and that had reverted to the wild in previous seasons, seeking them out and hand-feeding them to encourage these erstwhile human companions to stay close to socialize and play. Unsocialized, free-ranging wolves attracted to the area may have scavenged from the mammoth kill sites once humans abandoned them. These were located some distance from human camps.

Under this scenario, pups born at the camp sites or in liminal dens in these areas could have descended from lineages that had been subjected to many generations of unconscious human selection for particular morphological and behavioral traits, such as characteristics associated with tameness (e.g., increased sociability and playfulness). It therefore seems plausible that many pups taken from these dens could have displayed altered craniodental morphometry and body size reduction, resulting in the distinctive Palaeodog morphotype (Germonpré et al., 2009). Indeed, in the model proposed here, young socialized Palaeodogs probably would have been the canid type that was most commonly encountered in and around the large congregation sites, either as camp-living companions or in loose association with people (see also Koungoulos, 2021); both variants could have survived principally on food provided by humans (i.e., lean meat and bones from reindeer and muskoxen; Bocherens et al., 2015; Prassack et al., 2020, 2021).

Palaeodogs that had been hand-reared and socialized may still have been predisposed to disperse from human communities when they matured, forming mated pairs with socialized or unsocialized wolves, or socialized or unsocialized Palaeodogs – and giving birth in camp, in liminal dens, or further away in wild dens. Some of the dispersing socialized canids (both Palaeodogs and wolf types) may have roamed beyond the liminal zone into areas where they came into direct contact with packs of free-living, unsocialized wolves, forming mated pairs and interbreeding with them. Furthermore, humans gathered at the seasonal camps might have acquired some wolf pups from dens located further out in the non-anthropogenic or “wild” environment (perhaps because competition for pups from liminal dens was intense), bringing them back to camp to raise alongside pups born in the camps or obtained from the liminal zone (Figure 8).

### Breeding in camp

Some Palaeodog lineages, having acquired more behavioral traits associated with domestication, may have been inclined to freely come and go from human camps as adults, or even to reside in them on a permanent or semi-permanent basis. Concerning the latter, it seems likely that the liminal zone was not a completely safe area for socialized canids that had dispersed from human communities. Dangerous predators such as cave hyaenas, cave lions, and brown bears could also have been visiting this zone and may have preyed on them. Some socialized adult canids could have managed in this environment, and hence their liminal dens could have been raided by humans. Others, however, were restrained to campsites or being flexible enough, had the ability to breed in the presence of humans and may have chosen to birth their pups inside the confines of hunter-gatherer campsites, where they were protected and cared for by humans and thus free from inter- and intra-specific competition (Germonpré et al., 2020, 2021a,b).

Even if so, however, the dingo model implies that Upper Paleolithic humans would have continually replenished (or augmented) their supply of wolf pups by raiding dens in the spring. As with dingoes, socialized adult wolves that remained in camp may have reproduced at a low rate in captivity. However, even if camp-living wolves *did* furnish humans with an adequate supply of new pups, the practice of raiding wild dens for more pups may have continued for non-utilitarian reasons. As the Yolngu example noted above suggests, perhaps den raids were still undertaken because they reinforced a perceived spiritual connection between humans and wild wolves. Whatever the case, we contend that the practice of den raiding and wild pup-raising was a long-term cultural tradition over large parts of Eurasia that was not confined to the earliest phases of dog domestication (Germonpré et al., 2012:195), as could be tacitly implied in the human-initiated model.

Such a complex human-canid relationship could have endured in different regions in parallel for a long period of time, slowly modifying the genes of the local wolf populations and leading to the development and retention of traits associated with domestication. However, the influx of wild wolf genes would account for the long road to genetic isolation. During den raids, moreover, it may not have been possible for humans to reliably distinguish between pups from the liminal dens and those from the wild dens. This would have permitted gene flow from the wild, thereby delaying reproductive isolation in socialized camp wolves as there were several possible sources of genetic input.

In sum, the onset of springtime aggregation by large groups of Upper Paleolithic hunter-gatherers may have initiated more pronounced genetic changes in the wild-living wolf population from which humans obtained their pups, changes that enabled people to more successfully keep the socialized adults in camp after they matured sexually. However, in-camp reproduction was probably a “work in progress.” Socialized canids would have frequently dispersed when they reached breeding age and the ongoing practice of den raiding in liminal zones meant that there was constant gene flow from the wild into any populations of camp-born canids (Figure 8).

### The transition to domestication

Many Palaeodog lineages that emerged at or in the vicinity of human aggregation sites may have gone extinct. At least one lineage, however, if isolated reproductively for long enough (hundreds or thousands of years), could have given rise to the ancestors of modern domestic dogs. But this need not imply that dogs were domesticated from wolves in the central or eastern European region, or that the mammoth megasites on the Moravian Gate or Russian Plain *per se* are directly related to the beginnings of dog domestication. It could be the case, for example, that wolves were domesticated somewhere in Central or East Asia (e.g., Gojobori et al., 2021; Bergström et al., 2022; D’Huy, 2022). Even if so, we anticipate that the earliest domestic dog lineages emerged in similar circumstances to those outlined here.

In Australia, the special relationship between Aboriginal people and dingoes seems to have existed over a long period of time without resulting in the domestication of these wild-living canids (Brumm, 2021); although, as previously indicated, the situation in the southeastern region of the mainland may have been more complicated. So why was the situation different in Late Pleistocene Eurasia? How did a close and long-term dingo-like association between humans and wolves transform into a domestic one? What changed? Was there a threshold that was passed? How the final transition to a more controlled breeding occurred is uncertain, but it may be related to an increasing human population density which could have led to larger social networks and trading systems, furnishing people with more opportunities to exchange pups. This would have reduced the need to raid dens for newborn pups and resulted in a more profound genetic isolation between the emerging dogs and the Pleistocene wolves. Presumably, at a certain point the behavioral characteristics of the protodogs, descending from more isolated lineages, were becoming obviously advantageous compared to those of the socialized wolves, such that their owners took more effort to keep them separated from the wolves or culled hybrid pups.

## Conclusion

The close relationship between Aboriginal Australians and wild dingoes, as documented in the ethnohistorical record, has implications for our understanding of the human-canid relations that may ultimately have given rise to domestic dogs in Late Pleistocene Eurasia. Based on the dingo analogy, we contend that regular raiding of wolf dens for pre-weaned pups was a defining trait of the intimate relationship between humans and wolves that long preceded the appearance of the earliest uncontested dog remains in Eurasia (~17 ka). We further infer that: (1) when hand-reared, socialized, human-selected wolf pups reached sexual maturity they often would have reverted to the wild to breed, although some could have mated in captivity; (2) the dispersing socialized wolves commonly formed mated pairs with each other, but sometimes with wild Pleistocene wolves; the former would have most commonly established their territory in a liminal zone situated between human settlement sites (anthropogenic habitat “islands”; *cf.* Spengler, 2022) and the environment inhabited by strictly wild-living wolves, which had a natural fear of humans and actively avoided them; (3) consequently, the socialized canids often denned and whelped in the liminal zone close to human camp sites and hence each new generation of wild-born pups humans took from dens located close to their camps would include some (or many) of the offspring of human-selected canids.

In order for human selection during the pup adoption process to alter the wolf gene pool it would have been necessary for the dispersing socialized wolves to reuse the same dens and for humans to raid these dens regularly (i.e., during successive wolf denning and whelping seasons), as proposed by Mech and Janssens (2022). We believe such criteria would have been met at seasonal aggregation sites in Upper Paleolithic Eurasia, such as the enigmatic mammoth megasites inhabited by foraging communities during the spring when wolves were birthing nearby. In such areas, a long-standing human tradition of raising wild-caught pups taken from dens in the liminal zone could have altered wolf populations from a genetic standpoint, giving rise to novel behaviors and morphological characters associated with the effects of domestication – hence, the relatively large number of anatomically distinct dog-like remains found at these megasites. Regular input of wild wolf genes, however, would have slowed down and complicated this process (Figure 8), which likely had many dead ends, until genetic isolation of one or a few protodog lineages led to the emergence of the first domesticated dogs.

## Data availability statement

The original contributions presented in the study are included in the article; further inquiries can be directed to the corresponding author.

## Author contributions

All authors listed have made a substantial, direct, and intellectual contribution to the work and approved it for publication.

## Conflict of interest

The authors declare that the research was conducted in the absence of any commercial or financial relationships that could be construed as a potential conflict of interest.

## Publisher’s note

All claims expressed in this article are solely those of the authors and do not necessarily represent those of their affiliated organizations, or those of the publisher, the editors and the reviewers. Any product that may be evaluated in this article, or claim that may be made by its manufacturer, is not guaranteed or endorsed by the publisher.
